# Supplementary material for: Role and mechanism of NCAPD3 in promoting malignant behaviors in gastric cancer
Source: Front Pharmacol. 2024 Apr 22;15:1341039. doi: 10.3389/fphar.2024.1341039 (PMC11070777; doi:10.3389/fphar.2024.1341039)
Supplement: Supplementary file 11 [file DataSheet2.ZIP › GSEA/GO_CC/my_analysis.Gsea.1599461408869/GO_SITE_OF_POLARIZED_GROWTH.html]

Details for gene set GO\_SITE\_OF\_POLARIZED\_GROWTH[GSEA]

|  || Dataset | filtered\_dataset.sample\_info.cls#WT\_versus\_NCAPD3\_MUT |
| Phenotype | sample\_info.cls#WT\_versus\_NCAPD3\_MUT |
| Upregulated in class | WT |
| GeneSet | GO\_SITE\_OF\_POLARIZED\_GROWTH |
| Enrichment Score (ES) | 0.359726 |
| Normalized Enrichment Score (NES) | 1.5011687 |
| Nominal p-value | 0.072438166 |
| FDR q-value | 0.6426921 |
| FWER p-Value | 0.979 |
Table: GSEA Results Summary

  

Fig 1: Enrichment plot: GO\_SITE\_OF\_POLARIZED\_GROWTH      
 Profile of the Running ES Score & Positions of GeneSet Members on the Rank Ordered List

  

| SYMBOL | TITLE | RANK IN GENE LIST | RANK METRIC SCORE | RUNNING ES | CORE ENRICHMENT || 1 | 323 | APBB2 | 35 | 0.977 | 0.0610 | Yes |
| 2 | 56288 | PARD3 | 99 | 0.790 | 0.0853 | Yes |
| 3 | 5967 | REG1A | 100 | 0.788 | 0.1548 | Yes |
| 4 | 56104 | PCDHGB1 | 115 | 0.781 | 0.2136 | Yes |
| 5 | 50861 | STMN3 | 126 | 0.777 | 0.2749 | Yes |
| 6 | 7248 | TSC1 | 158 | 0.718 | 0.3160 | Yes |
| 7 | 54434 | SSH1 | 183 | 0.692 | 0.3597 | Yes |
| 8 | 23242 | COBL | 426 | 0.516 | 0.2313 | No |
| 9 | 10280 | SIGMAR1 | 655 | 0.399 | 0.1025 | No |
| 10 | 57679 | ALS2 | 656 | 0.399 | 0.1377 | No |
| 11 | 23064 | SETX | 657 | 0.398 | 0.1728 | No |
| 12 | 23767 | FLRT3 | 781 | 0.333 | 0.1137 | No |
| 13 | 81544 | GDPD5 | 961 | -0.381 | 0.0186 | No |
| 14 | 23046 | KIF21B | 1034 | -0.433 | 0.0050 | No |
| 15 | 80315 | CPEB4 | 1053 | -0.448 | 0.0315 | No |
| 16 | 5818 | PVRL1 | 1073 | -0.458 | 0.0583 | No |
| 17 | 283 | ANG | 1074 | -0.458 | 0.0987 | No |
| 18 | 26053 | AUTS2 | 1113 | -0.485 | 0.1141 | No |
| 19 | 7074 | TIAM1 | 1143 | -0.508 | 0.1381 | No |
| 20 | 2043 | EPHA4 | 1228 | -0.602 | 0.1308 | No |
Table: GSEA details [plain text format]

  

Fig 2: GO\_SITE\_OF\_POLARIZED\_GROWTH      
 Blue-Pink O' Gram in the Space of the Analyzed GeneSet

  

Fig 3: GO\_SITE\_OF\_POLARIZED\_GROWTH: Random ES distribution      
 Gene set null distribution of ES for **GO\_SITE\_OF\_POLARIZED\_GROWTH**
